# The longitudinal influence of the level of executive function development on children’s transcriptional skills: a modern view of A. Luria’s ideas

**DOI:** 10.3389/fpsyg.2023.1199683

**Published:** 2023-06-07

**Authors:** Ekaterina S. Oshchepkova, Arina N. Shatskaya, Maria S. Kovyazina

**Affiliations:** ^1^Department of Educational Psychology and Pedagogy, Lomonosov Moscow State University, Moscow, Russia; ^2^Psychological Institute of the Russian Academy of Education, Moscow, Russia

**Keywords:** cultural-historical theory, writing skills, executive functions, transcriptional skills, writing development

## Abstract

In the realm of Cultural-Historical Theory, A. Luria suggested writing as a model of a complex system of higher mental function, since that function is based on various psycho-physiological mechanisms, including processing of audial and visual information; and as a complex system of the frontal lobe functions of overcoming perseveration, and creation and control of the writing program. Subsequent research on these topics has shown a close association between the level of development of executive functions (EF) and writing skills. Nevertheless, the question of which parts of EF influence which aspects of writing, remains unresolved. In addition, there are few longitudinal studies of EF’s influence on writing. In this article, we focus on the results of a longitudinal study of the influence of EF in children 6.3 years old on their mastery of basic writing skills at the age of 7.5. The results of regression model construction showed that all the aspects of executive functions strongly influenced the children’s transcription skills, while the greatest impact on the development of the graphomotor component and spelling skills was exerted by working memory and inhibition control. These results are consistent with studies that have shown a correlation between the development of writing skills and EF. These results also confirm A. Luria’s views on the importance of functions responsible for processing audial and visual information in the process of writing, and the importance of suppressing irrelevant stimuli and perseverations. Our research shows the importance of the development of EF in preschool childhood.

## 1. Introduction

The first of Luria’s works about the development of writing in children appeared in 1929. It was “Voprosy marksistkoi pedagogikii [Problems of Marxist education]. Moscow: Academy of Communist Education, 1929. Vol. 1, pp. 143–176” (cited in Luria, 1978). There he noted that “For a child to be able to write or note something, two conditions must be fulfilled. First, the child’s relations with the things around him must be differentiated (…). Second, the child must be able to control his own behavior by means of these aids, in which case they already function as cues he himself invokes” (Luria, 1978).

In 1950, A. Luria’s “Essays on the Psychophysiology of Writing” was published (Luria, 1950, 1978). In this work, he set the task of helping primary school teachers and specialists understand the process of writing and its organization from the standpoint of neuropsychology and psychophysiology. To do this, Luria used neuropsychological data on how writing skills are impaired when various parts of the brain are injured. Approaching writing as a higher mental function, A. Luria identified the following writing stages: (1) analysis of phonemic word content; (2) translation of highlighted phonemes into a graphic form; and (3) transformation of optical signs into necessary graphic outlines. When Luria discussed the mechanisms behind these stages, he emphasized the roles of the auditory analyzer (unlike the usual writing process), articulation, and visual organization of the writing process, as well as the importance of the frontal lobes in planning tasks and suppressing unnecessary activity and perseverations.

Further development of Luria’s ideas in the school of cultural-historical psychology has revealed that writing skills, as a part of the general notion of literacy, are an important aspect of children’s mental development, and a condition for successful schooling (Solovieva et al., 2021; Veraksa and Veraksa, 2021). Now Luria’s followers among speech therapists and neuropsychologists continue to apply his ideas to overcome learning disabilities in children (Velichenkova et al., 2001; Akhutina, 2004).

The expanding development of electronic communication is leading to children typing on keyboards more often than writing by hand; this practice deprives them of the necessary prerequisites for developing writing skills (Mayer et al., 2020), which is becoming a greater problem of its own.

Writing as a set of rules, and the skill of capturing certain meanings in written form, requires the development of transcription skills (handwriting and spelling) (Rocha et al., 2022). Transcription skills (handwriting and spelling) may be assessed by various methods. We used methods developed by the Vygotsky-Luria school, which are currently successfully identifying children’s learning disabilities (Akhutina and Pylaeva, 2012; Glozman and Plotnikova, 2021).

The connection between EF and transcription skills has been studied (Hayes and Berninger, 2014; Yeung et al., 2017). However, the question remains as to which aspects of EF influence handwriting the most, and which influence spelling. Moreover, the number of longitudinal studies dedicated to the influence of EF on transcription skills are few. This fact explains the novelty of our research: what is the connection between different aspects of children’s EF and the development of their writing skills in the Russian language, as they use Cyrillic writing with its predominance of orthography and morphology in spelling (Boulware-Gooden et al., 2015).

We posed the following research questions in our study: (1) Which EF aspect has the greatest connection to the productivity of a child’s graphomotor test performance; and (2) Which aspect of EF is the most connected to the level of development of spelling skills?

## 2. Method

Our research sample consisted of children living in Moscow. We have selected the children from 6 to 7 y.o. because we are interested first of all in the process of child’s transition from preschool to school and in the changes that occur during this process. Two meetings, with an interval of approximately 1 year in-between, were conducted with them. At the first stage of the research, the children were attending kindergarten (*n* = 346, *M* = 6.24 y.o., SD = 4.15 mth), while at the second stage, the same children were attending first grade. The number of children had decreased (*n* = 271, *M* = 7.5 y.o., SD = 6.18 mth). So, the final size of the sample was 271 persons (100 boys and 171 girls). During both stages, the children’s level of development of the components of executive functions was diagnosed (verbal and visual working memory, cognitive flexibility, inhibitory control). In the second stage of diagnostics, an assessment of the children’s writing skills development was also conducted. According to parents’ questionnaire their social cultural level was middle or high-middle. The teaching methods corresponded to a typical educational program in Russia for monolingual regions. According to this program the children begin to study written transcription only in primary school. So when the study took place, the children had been studying written transcription for 7 months.

Executive functions (EF) is a very complex concept with different approaches to it. We followed Miyake’s model of EF (Miyake et al., 2000). The NEPSY-II complex (Korkman et al., 2007) was used to assess almost all aspects of the children’s EF. However, the evaluation of cognitive flexibility for school students and preschoolers was conducted by different methods due to age restrictions specified by the authors. So, for younger school students, a task from NEPSY-II was used, and for preschoolers, the *“DCCS”* (Zelazo, 2006) method was applied.

Moreover, the “Raven’s progressive matrices” (Raven and Court, 1998) test was applied in order to control the factors of individual differences in intellectual development. Only the children with normative cognitive development participated in further study.

The diagnosis of the children’s transcription skills was conducted based on the following tests (Akhutina and Pylaeva, 2012): (a) the graphomotor test: the child is asked to write a number of alternating elements without raising their hand from the paper (the correctness of task performance is assessed); (b) the child is asked to write down their name and surname (the writing correctness is assessed); (c) the child is asked to write all the block letters they know, without repeating them (the total amount of letters written correctly without repetitions and the total number of mistakes made are assessed); (d) the child is asked to write down six syllables from dictation (the total writing correctness and the number of mistakes made are assessed); (e) the child is asked to write down three short sentences from dictation (the writing correctness, the number of words missed, the number of spelling and other mistakes – i.e., merged spelling of words and sentences, letter omission, incorrect use of upper/lower case letters, etc. -- are assessed); and (f) the child is asked to look at a number of purposefully incorrectly written words and correct the mistakes (the number of corrected words and correction mistakes made by the child are assessed).

As a final indicator, the following parameters were measured: (1) the productivity of graphomotor test performance; (2) spelling skills: and (3) the overall productivity of task performance from tests b-f above.

The data obtained was analyzed in the following way:

1. In the first testing (at 6 y.o.), we divided the sample into three groups according to the children’s levels of EF development: high, average, or low. Then we calculated the correlations between the levels of development of all EF components at 6 years of age and the levels of development of the highlighted writing skills indicators 1 year later. In particular, we analyzed the connections between the level of writing skills at the age of 7 with the level of the following parameters at the age of 6: (a) verbal and visual working memory; (b) cognitive flexibility; and (c) inhibitory control.
2. We constructed a general linear model. We chose the writing skills of the 7-year-old children as our dependent variable. As predictors, we selected the scores on all EF functions recorded at 6 years of age: audial and visual working memory; inhibitory control; and cognitive flexibility. Moreover, our model included such individual factors as gender, age (measured in months), and level of intellectual development (estimated with Raven’s progressive matrices).

## 3. Results

### 3.1. Predictors of graphomotor writing skills development

#### 3.1.1. Working memory influence

First, we compared the graphomotor test indicators among the children with low, average, and high levels of verbal and visual working memory. The results showed that the three groups differed distinctively in regard to both kinds of working memory (*F* = 7.93, *p* < 0.001 and *F* = 7.75, *p* < 0.001, accordingly). So, children with high levels of visual and verbal working memory at the age of 6 showed the highest scores in graphomotor test performance (*M* = 4.15, SD = 0.81 for visual memory, *M* = 4.2, SD = 0.76 for verbal memory), while children with low levels of working memory showed the lowest performance scores (*M* = 3.59, SD = 0.87 for visual memory, *M* = 3.58, SD = 0.89 for verbal memory). Thus, the higher the level of verbal and visual working memory development in preschool, the better the children were at performing the graphomotor test in the first grade of school.

#### 3.1.2. Inhibitory control influence

Second, we compared the graphomotor test indicators for children age 7, who had previously (1 year ago) demonstrated low, average, and high levels of inhibitory control development. The results indicated that all three groups were distinctively different (*F* = 6.55, *p* = 0.002).Thus, the children with a low level of inhibitory control at the age of 6, showed lower levels of graphomotor test performance (*M* = 3.63, SD = 0.89), while children with higher levels of inhibitory control showed significantly better results (*M* = 4.14, SD = 0.78).

#### 3.1.3. Connection to cognitive flexibility

The analysis showed that there was no significant difference between children with low, average, and high levels of cognitive flexibility (*F* = 2.15, *p* = 0.121).

Thus, the level of graphomotor test performance at the age of 7 was significantly associated with the level of verbal and visual working memory, as well as the inhibitory control level at the age of 6 years old. A similar association, although not as statistically significant, can be noted in regard to cognitive flexibility. The more developed these EF components were at the age of 6, the better the children were at graphomotor test performance at the age of 7. The level of writing skills development depended on the level of self-regulation while still in kindergarten.

We note that the following indicators were used in the scoring of integral spelling skills mastery: (1) the productivity of writing one’s own name and surname; (2) writing alphabet letters; (3) writing syllables and sentences under dictation; and (4) the test of correction of word mistakes. We present the results obtained on each component in detail, with the results of descriptive statistics on the parameters of integral spelling skills mastery, as well as its five components (see Table 1).

**Table 1:** Descriptive statistics on spelling skills indicators at 7–8 years old.

	Integral indicator, spelling skills (%)	Name-surname	Alphabet	Syllables	Sentences	Word mistakes correction
Mean	86.1	4.77	21.3	5.74	12.1	8.16
Median	88.2	5	24	6	13	8
Standard deviation	10.9	0.471	10.6	0.799	1.88	1.78
Min	21.3	3	0	0	0	0
Max	100	5	33	6	13	10

Moreover, we calculated the correlations between mistakes made in all the writing tests at 7 years of age, and the level of development of all EF components prior to that, when the children were age 6. The results revealed that all significant connections between the mistakes made in all the writing tests and EF were of negative coefficients (i.e., the higher the EF indicator, the fewer mistakes the children made). However, this was not the case for the alphabet writing test (*r* = 0.199, *p* = 0.002). The most potent association was between the overall number of mistakes made in the sentences writing test and the level of verbal working memory development. The more developed the verbal working memory, the fewer mistakes the children made.

### 3.2. Predictors of spelling skills development

Regarding the relationship between the level of EF development at the age of 6 and spelling skills development, the following results were obtained:

#### 3.2.1. Working memory

**Figure 1:**
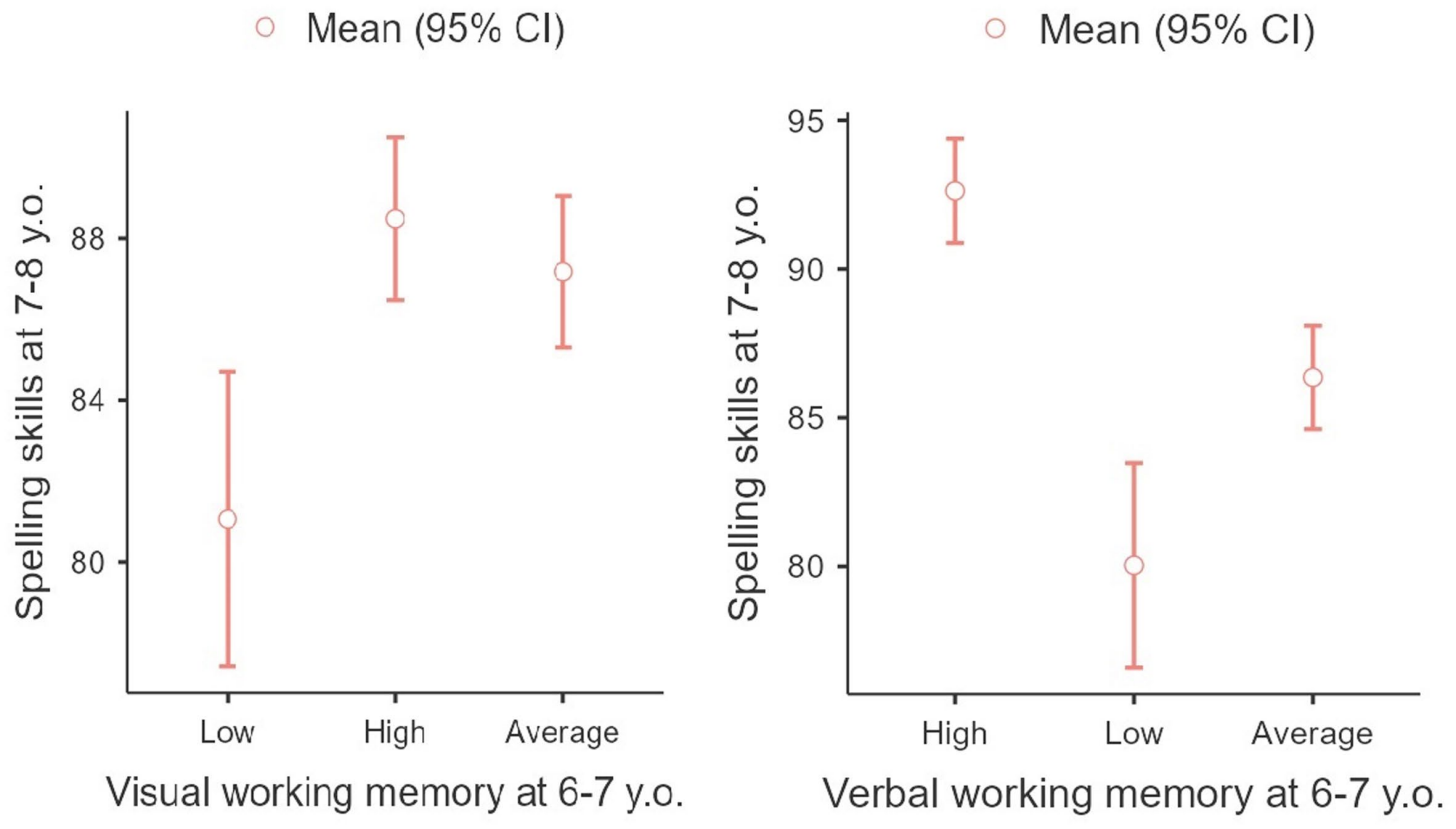
Connection between spelling skills mastery at the age of 7–8 y.o. with working memory at the age of 6–7 y.o.

The results showed that the separate groups significantly differed in accordance with every type of working memory (*F* = 6.38, p = 0.002 and *F* = 26.1, *p* < 0.001). Thus, children with high levels of visual and verbal working memory at the age of 6 demonstrated a considerably higher level of spelling skills a year later, while children with low levels of development of these components of EF showed a lower level of spelling skills (see Figure 1).

#### 3.2.2. Inhibitory control

**Figure 2:**
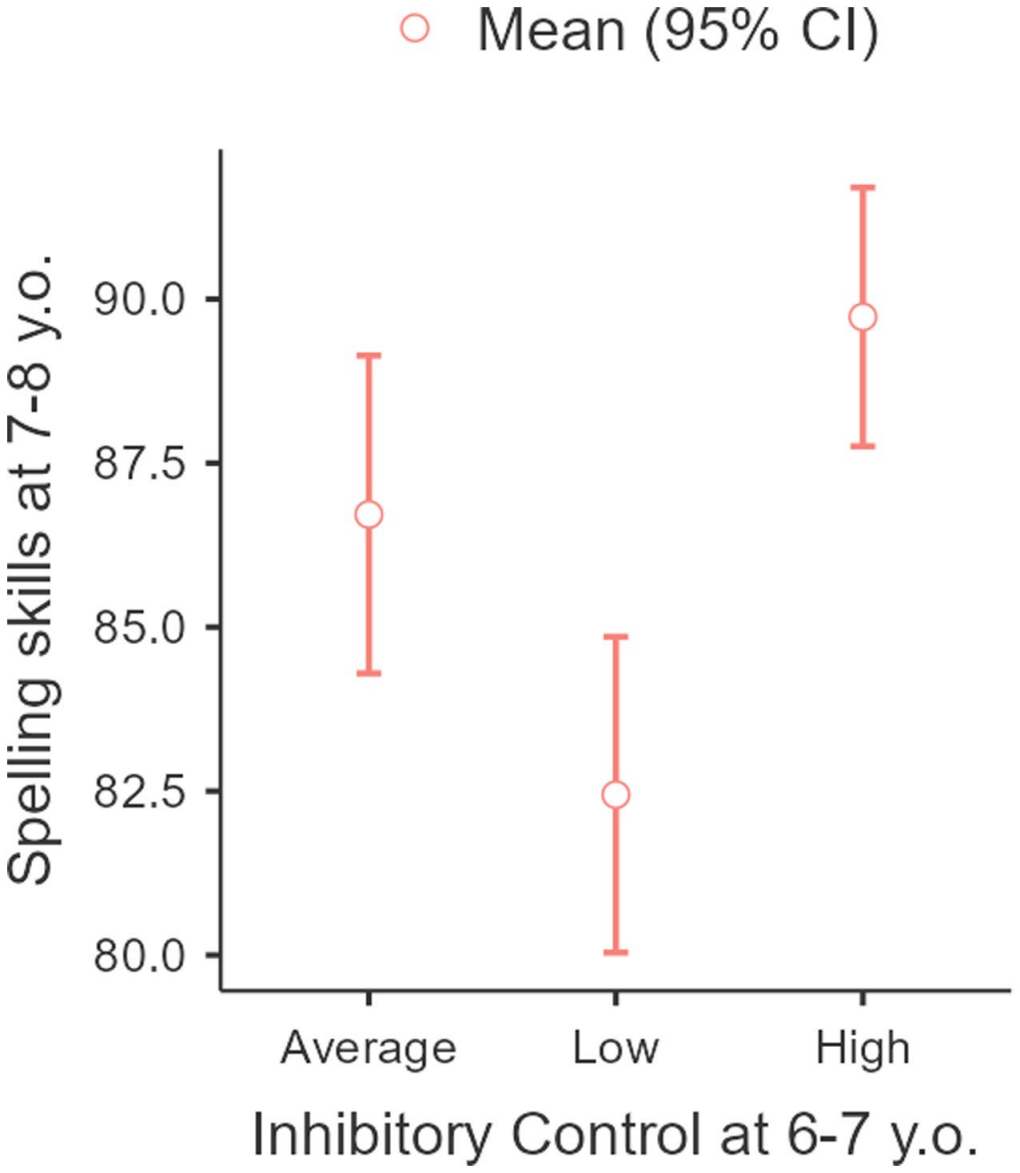
Connection between spelling skills mastery at the age of 7–8 years old with inhibitory control at the age of 6–7 years.

It has been shown that there were significant differences between certain groups of children in this area (*F* = 10.8, *p* < 0.001). Children from the “high” inhibitory control group (at the age of 6) demonstrated a higher level of spelling skills mastery at age 7 (see Figure 2).

#### 3.2.3. Cognitive flexibility

**Figure 3:**
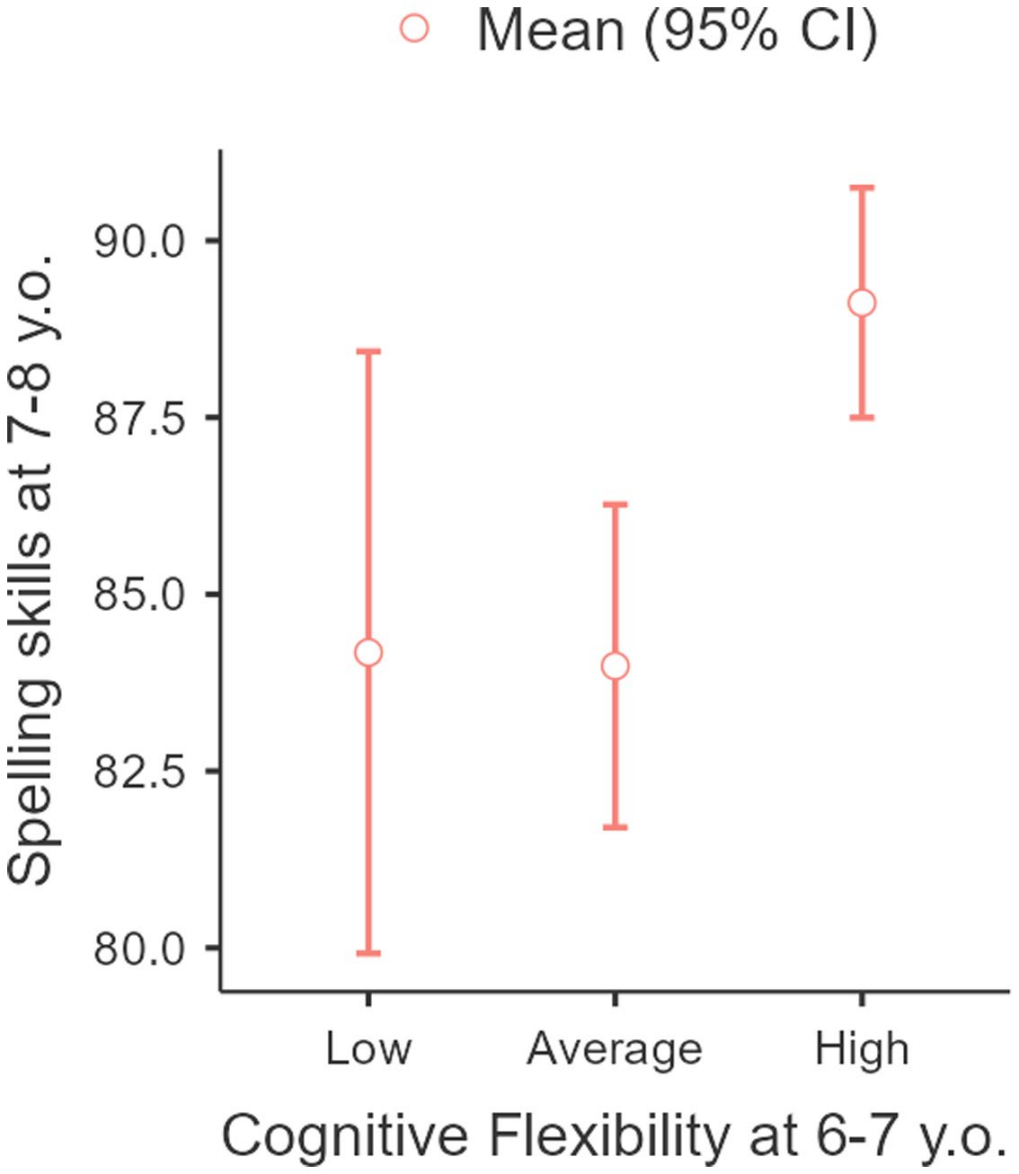
Connection between spelling skills mastery at the age of 7–8 years old with cognitive flexibility at the age of 6–7 years old.

Significant differences between the groups with high, average, and low levels of cognitive flexibility were shown (*F* = 7.61, *p* < 0.001). A year later, the children with a high level of cognitive flexibility demonstrated a high level of spelling skills mastery, while the children with average and low levels of cognitive flexibility show those same levels of spelling skills mastery (see Figure 3).

**Figure 4:**
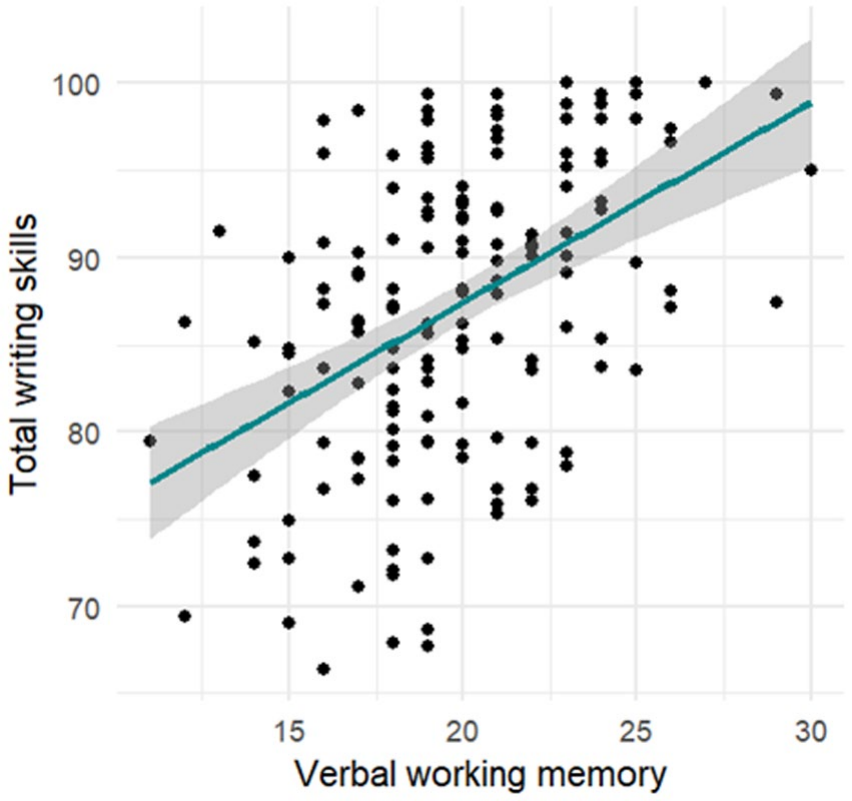
Connection between spelling skills mastery at the age of 7–8 years old with audial working memory at the age of 6–7 years old.

After editing out the missing values and checking the basic assumptions for linear model construction, we found that the model is homoscedastic. Therefore, we decided to use the weighted least squares regression method. The predictors did not demonstrate multicollinearity (VIF < =1.519). The remaining regression assumptions were also successfully checked. As a result of the stepwise removal of the non-significant predictors, the final model consisted of the following predictors: (a) the features of intellectual development; (b) inhibitory control; and (c) verbal working memory (*F* = 23.32, *p* < 0.000, Adjusted R-squared: 0.2925). After comparing the regression estimate predictors in the final model, it turned out that the most important contribution to the total spelling skills score at the age of 7 was made by the level of verbal working memory at the age of 6 (see Figure 4). The second most important impact was exerted by inhibitory control, and the third greatest by level of intellectual development.

Thus, all the components of EF at an earlier age were connected to the level of spelling skills mastery at the age of 7. However, the most important contributions were made by verbal working memory and inhibitory control.

## 4. Discussion and conclusion

A. Luria demonstrated that the participation of audial and visual analyzers, as well as the brain’s frontal lobes, is crucial for a successful development of the writing process.

As is shown by neuro-visualization studies, verbal working memory is connected to the same brain sections as the audial analyzer, as Luria stated (Kumar et al., 2016), while visual working memory is connected to the same brain sections as the visual analyzer, as also described by Luria (Ungerleider et al., 1998). In addition, inhibitory control is connected to the brain’s frontal lobes (Knyazev, 2007). This is why the data we obtained from a longitudinal population sample of normally developing children confirms the results which A. Luria found in studies of adults with brain dysfunctions.

The research also confirms the results of studies of the connection between EF and writing (Cordeiro et al., 2020) over the long run, with consideration of the specifics of writing in the Russian language.

In regard to the specific aspects of transcriptional skills, according to our research, working memory and inhibitory control influence the graphomotor skills first and foremost.

We can assume that the connection to working memory is based on the fact that good memorization of samples and instructions (visual and verbal memory) contributes to correct performance during this test. As for inhibitory control, the significant aspect of the graphomotor test is the withholding of stereotypical hand movements, as the child is trying to draw straight or tilted lines only, without interchanging them. Inhibitory control allows the child to inhibit irrelevant hand movements and continue with the correct execution of the task.

The level of spelling skills (in the first grade of school) was influenced by all the EF components in the school preparatory kindergarten group. We assume that this could be explained by how verbal memory allows the children to retain words and sentences they were given in dictation, while inhibitory control allows them to use spelling rules, instead of writing down words in the way they are heard.

To summarize, our research on a sample of children writing in a Cyrillic system with its predominance of orthography and morphology in spelling performance (Boulware-Gooden et al., 2015) has confirmed the earlier studies of the connection between executive functions and the writing process (Limpo and Olive, 2021).

Our study allows us to formulate a number of recommendations for educators, especially those preparing children for school or working to overcome learning difficulties. We can state that the development of executive functions will have a significant positive impact on the development of literacy in children.

Our study has a number of limitations. First, it was carried out on monolingual children. We assume that bilingual children will have their own characteristics in mastering transcription skills, which is associated, on the one hand, with better development of regulatory functions, and on the other hand, with possible difficulties in mastering different writing systems (for example, Latin and Cyrillic). Secondly, it included only middle and high-middle class children with normative development. It is possible that other patterns will be revealed in children with social or cognitive difficulties. Thirdly, in contrast to the studies of A. Luria, which were conducted on the basis of neurological data, our study relies on a non-clinical approach through the development of executive functions. In order to draw unambiguous conclusions, it would be good to compare the Luria’s and Miyake’s concepts and their contribution to the development of writing.

## Data availability statement

The raw data supporting the conclusions of this article will be made available by the authors, without undue reservation.

## Ethics statement

The studies involving human participants were reviewed and approved by the Ethics Committee of the Faculty of Psychology at the Lomonosov Moscow State University (the approval no: 2021/98). Written informed consent to participate in this study was provided by the participants’ legal guardian/next of kin.

## Author contributions

MK was responsible for the methodological basis and understanding of the theoretical and practical significance of the research. EO was responsible for the design development, defined the diagnostic tools, and wrote the individual parts of the text of the manuscript. AS was responsible for the data collection, analysis of the received data, and wrote the individual parts of the text of the manuscript. All authors contributed to the article and approved the submitted version.

## Funding

This study was part of the research project “The factors and effects of oral and written speech development in 6-8-year-old children in mono- and bilingual environment: a longitudinal study,” work was supported by the Russian Science Foundation grant number 21-18-00581.

## Conflict of interest

The authors declare that the research was conducted in the absence of any commercial or financial relationships that could be construed as a potential conflict of interest.

## Publisher’s note

All claims expressed in this article are solely those of the authors and do not necessarily represent those of their affiliated organizations, or those of the publisher, the editors and the reviewers. Any product that may be evaluated in this article, or claim that may be made by its manufacturer, is not guaranteed or endorsed by the publisher.
